# Computational Promoter Modeling Identifies the Modes of Transcriptional Regulation in Hematopoietic Stem Cells

**DOI:** 10.1371/journal.pone.0093853

**Published:** 2014-04-07

**Authors:** Sung-Joon Park, Terumasa Umemoto, Mihoko Saito-Adachi, Yoshiko Shiratsuchi, Masayuki Yamato, Kenta Nakai

**Affiliations:** 1 Human Genome Center, the Institute of Medical Science, the University of Tokyo, Tokyo, Japan; 2 Institute of Advanced Biomedical Engineering and Science, Tokyo Women's Medical University, Tokyo, Japan; B.C. Cancer Agency, Canada

## Abstract

Extrinsic and intrinsic regulators are responsible for the tight control of hematopoietic stem cells (HSCs), which differentiate into all blood cell lineages. To understand the fundamental basis of HSC biology, we focused on differentially expressed genes (DEGs) in long-term and short-term HSCs, which are closely related in terms of cell development but substantially differ in their stem cell capacity. To analyze the transcriptional regulation of the DEGs identified in the novel transcriptome profiles obtained by our RNA-seq analysis, we developed a computational method to model the linear relationship between gene expression and the features of putative regulatory elements. The transcriptional regulation modes characterized here suggest the importance of transcription factors (TFs) that are expressed at steady state or at low levels. Remarkably, we found that 24 differentially expressed TFs targeting 21 putative TF-binding sites contributed significantly to transcriptional regulation. These TFs tended to be modulated by other nondifferentially expressed TFs, suggesting that HSCs can achieve flexible and rapid responses via the control of nondifferentially expressed TFs through a highly complex regulatory network. Our novel transcriptome profiles and new method are powerful tools for studying the mechanistic basis of cell fate decisions.

## Introduction

Hematopoiesis is a complex and dynamic process, which generates mature blood cells throughout the life of organisms. In the adult bone marrow, long-term hematopoietic stem cells (LT-HSCs) maintain a balanced pool of stem cells, which also differentiates into more mature short-term hematopoietic stem cells (ST-HSCs), multipotent progenitors with a lower self-renewal capacity. It is believed that the blood lineage choice of HSCs is governed by a stepwise cell fate decision [1], [2]. However, recent studies have raised questions about the hierarchical hematopoietic system [3], [4]. Many studies based on genome-wide gene expression profiling [5]–[9] have demonstrated that specific extrinsic and intrinsic regulators play key roles in hematopoiesis [10]–[12]. Recently, high-throughput sequencing techniques have been applied widely [13]–[15], which have provided new insights into *in vivo* transcription factor (TF) binding and epigenetic modifications [16]–[18]. Systems biology approaches are also enhancing our understanding of the regulatory dynamics of hematopoiesis [19].

Despite the biological importance of the formation of all blood cells via a transition from LT-HSC to ST-HSC, little is known about the mechanism that underlies this early differentiation. A major explanation for this deficiency is a lack of comprehensive genome-wide identification studies and characterizations of the regulatory elements that govern gene expression in HSCs. The profiling of potential key regulators [8], [17], [20] and the large-scale integration of datasets [21], [22] have improved our understanding greatly. However, these studies are limited to a small number of factors that function in heterogeneous HSCs, which were isolated using different combinations of monoclonal antibodies. Therefore, unconsidered key regulators may exist at this early stage of hematopoiesis. Indeed, novel key factors [23], [24] and new multipotent progenitors [3], [4], [25] have been identified recently.

To address these deficiencies, we developed a computational method on the basis of novel transcriptome data from adult mouse bone marrow HSCs; 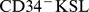 (c-kit^+^Sca1^+^Lin^−^) LT-HSCs and 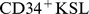 ST-HSCs, a widely used strategy to isolate HSCs at high purity [26], [27]. Our method uses a regression-based approach [28]–[30] to model the linear relationships between gene expression and the characteristics of regulatory elements compiled from a database. In the present study, we extended this regression modeling-based approach using large-scale log-linear modeling (LLM) [31], which considered the combinatorial nature of TFs. Thus, our method can systematically infer the regulation modes exerted by TFs that are probably necessary for gene expression, as well as suggesting synergistic TF modules. Using our transcriptome profiles and this novel method, we characterized transcriptional regulatory modes related to HSCs, which suggested the functional importance of TFs expressed at steady-state or low levels. Remarkably, we identified 24 differentially expressed TFs that targeted 21 putative TF-binding sites (TFBSs) in LT-HSCs. These TFs might be essential for maintaining the HSC capacity during the early stage of hematopoiesis.

## Results

### Extensive transcriptome discovery

#### RNA-seq analysis of HSCs

**Figure 1:**
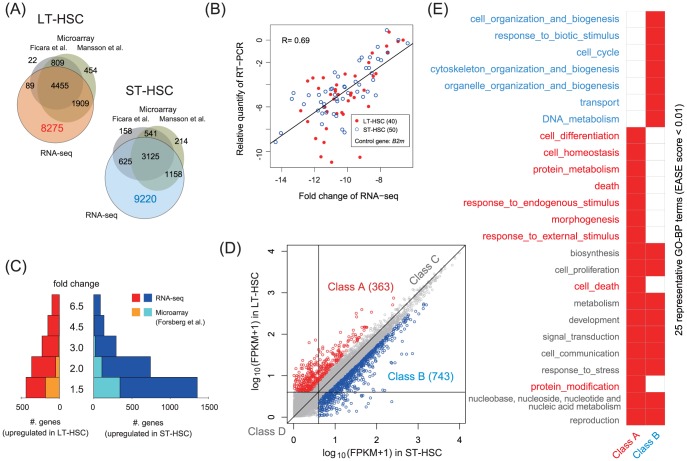
Extensive transcriptome discovery based on the RNA-seq assay. (A) Our RNA-seq assay discovered over 8200 mRNAs that were not detected in microarray-based studies. (B) RNA quantities relative to those of the housekeeping gene beta-2 microglobulin (*B2m*) were correlated in qRT-PCR and RNA-seq assays, but variations were also observed in genes that were expressed at low levels. (C) Analysis of gene expression changes detected a transcriptionally active state in ST-HSCs with a larger number of genes than those considered previously. (D) We categorized genes into 4 classes; Class A and Class B, in which FC 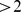 and FPKM 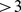, Class C (6332 genes), in which FC 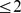 and FPKM 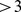, and Class D (6006 genes), in which FPKM 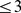. Class A and Class B represented DEGs, Class C represented steady-state transcription genes, and Class D represented genes with noisy expression and/or functional low-expression genes. (E) Enriched GO biological process (GO-BP) terms detected by DAVID (EASE score, 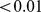, complete lists in Tables S10 and S11).

To establish transcriptional profiles, we extracted total RNA from mouse LT-HSCs (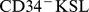) and ST-HSCs (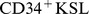), and performed SOLiD RNA-seq assays in triplicate. We generated 44–70 million 50 bp short reads, among which 44%–63% were mapped uniquely to the mouse genome (mm9) via our recursive mapping strategy [32]. These uniquely mapped reads (uni-reads) were used for further analysis (Table S1). We used the TopHat/Cufflinks pipeline [33] to quantify the RNA abundance of RefSeq genes as fragments per kilobase of exon per million mapped reads (FPKM). This analysis confirmed the high reproducibility among replicates (Figure S1A). We also assessed the overlap between our profile and public expression profiles [8], [9]. This comparison showed that our RNA-seq assay uniquely identified 8275 and 9220 genes from LT- and ST-HSCs, respectively (Figure 1A). This indicates that our study successfully identified a more detailed transcriptome landscape than previous studies.

The application of different monoclonal antibodies to purify HSC populations may have diverse effects on the resulting expression profiles [2], which are related to issues regarding the functional purification of HSCs [10], [26] and the heterogeneous expression in single cells [4], [10], [34]. In fact, a comparison between our findings and the results of an RNA-seq analysis of HSCs isolated using distinct markers [15] demonstrated that there were great differences, particularly among genes that were expressed at low levels (Figure S1B). In addition, we performed qRT-PCR using 90 genes that were randomly selected from our samples, and confirmed that RNA quantities relative to the housekeeping gene *B2m* were in overall agreement (Figure 1B). However, genes that were expressed at low levels were substantially different. These results suggest the difficulty in detecting and quantifying rare transcripts in HSCs.

#### Identification of differentially expressed genes (DEGs)

We identified genes with high expression levels (FPKM, 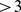) and calculated the fold change (FC) in gene expression. This analysis detected the transcriptionally active state of ST-HSCs (Figure 1C), which supported the results of previous studies [6], [7], [15]. Our RNA-seq assay detected a higher number of DEGs than those reported previously, which may have been related to our more comprehensive transcriptome discovery method. We categorized the genes into 4 classes using a change of 2-fold as the threshold [15] (Figure 1D): Class A, 363 genes upregulated in LT-HSC; Class B, 743 genes downregulated in LT-HSC; Class C, 6332 genes with FC 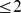 and FPKM 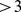; and Class D, 6006 genes with low expression (FPKM, 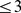). Thus, Class A and Class B represented DEGs, Class C represented steady-state transcription genes, and Class D represented genes with noisy expression and/or functional low expression genes.

We searched for any gene ontology (GO) terms enriched in DEGs using the DAVID Bioinformatics Resources [35]. Figure 1E shows the representative GO terms (Tables S10 and S11 for complete lists). This analysis showed that DEGs were involved in the immune response, cell–cell communication, and signal transduction. This was not surprising because extrinsic and intrinsic signals and molecules contribute to the biology of HSCs in the bone marrow microenvironment [1], [10], [11], [36]. In addition to these common biological processes, Class A genes were involved particularly in cell death, cell differentiation, and homeostasis, whereas Class B genes were involved in DNA repair, cell cycle progression, and cell organization. These results were consistent with those of previous studies that showed that apoptosis and cell-cycle regulators play critical roles in maintaining a balanced pool of HSCs and in the expansion of progenitor populations [5], [37], [38].

#### Differentially expressed cell-surface molecules and TFs

DEGs included 77 cell-surface molecules with the ''cell surface'' (GO:0009986) GO term (Table S2), some of which are known to be associated with hematopoiesis: in Class A, *Vwf*, *Lhcgr*, *Cxcl12*, and *Tgfbr3*; in Class B, *CD244*, *CD33*, and *Clec12a*. *CD34*, which was used to isolate HSCs in this study, exhibited an upregulation of over 12-fold in ST-HSCs compared with LT-HSCs. To obtain high HSC purities, these cell-surface molecules will be useful as alternative or additional markers.

DEGs also included 57 TFs that were annotated in TRANSFAC [39], i.e., 31 in Class A and 26 in Class B (Tables 1 and S3). These differentially expressed TFs included known hematopoietic regulators (e.g., *Gata2*, *Tal1*, and *Satb1*) and previously unconsidered TFs, such as the hepatocyte nuclear factor *Foxa3*, the BTB-domain zinc finger *Zbtb20*, the DNA-binding domain *Arid5a*, and the epigenetic regulator *Uhrf1*. It was noteworthy that a large number of TFs belonged to Class C (303 TFs) and Class D (341 TFs) (Tables S4 and S5). In particular, TFs with synergistic functions in HSCs [17] and that belonged to TF families, such as Fox, Lmo, and Sox (which are required by HSCs), were present in Class C and/or Class D. These results may suggest that, in addition to differentially expressed TFs, TFs with coding genes that are expressed at stable or low levels are functionally important molecules.

**Table 1:** Top ten differentially expressed transcription factors.

Class	Gene	FC*	Microarray†	Description
A	Rorc	6.4252		RAR-related orphan receptor gamma
	Hoxb5	5.1317		homeobox B5
	Rarb	3.8601		retinoic acid receptor, beta
	Cdkn1c	3.8479	M,Fo	cyclin-dependent kinase inhibitor 1C (P57)
	Fosb	3.0942	Fi,M	FBJ osteosarcoma oncogene B
	Car1	2.9839	M	carbonic anhydrase 1
	Id1	2.9708		inhibitor of DNA binding 1
	Klf1	2.8796	M	Kruppel-like factor 1 (erythroid)
	Nr4a1	2.7957	Fi,M	nuclear receptor subfamily 4, group A, member 1
	Foxa3	2.7509		forkhead box A3
B	Satb1	3.7749	Fi,M,Fo	special AT-rich sequence binding protein 1
	Hnf4a	3.1733		hepatic nuclear factor 4, alpha
	Trf	2.5921		transferrin
	Hmgb2	2.0842	M	high mobility group box 2
	Runx3	1.9827		runt related transcription factor 3
	Irf8	1.8349		interferon regulatory factor 8
	Arid5a	1.7884		AT rich interactive domain 5A (MRF1-like)
	Uhrf1	1.4536		ubiquitin-like, containing PHD and RING finger domains, 1
	Zfp422	1.4477	Fi,M	zinc finger protein 422
	Notch1	1.3403		notch 1

*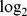 fold change.

† M: Mansson et al. [8], Fo: Forsberg et al. [6], Fi: Ficara et al. [9].

### Computational modeling of DEG promoters

#### Workflow overview of promoter modeling

To determine the upstream regulatory elements that are essential for DEG transcription, we used a linear regression model that was used widely for this purpose in previous studies [28], [30]. The underlying assumption of this model is that the expression levels of genes are controlled by the sum of the independent activities of regulators, such as DNA-binding factors or epigenetic marks. These activities can be approximated using high-throughput *in vivo* experiments [40], [41] or knowledge-based computational approaches [25], [30]. As a preliminary test, we applied the linear regression model described in our previous study [29] using ChIP-seq data for 10 major TFs [17]. In this approach, we used genome-wide TF-binding instances that occurred within 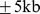 regions from transcription start sites (TSSs), and predicted the FPKMs of DEGs by using a simple linear regression model with rigorous statistical tests. However, we were unable to detect any significant effects, and the correlation between the observed and predicted FPKMs was 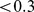. This failure may, in part, reflect the possibility that these TFs exert regulatory functions as distal enhancers, rather than through proximal promoters [17], [42].

**Figure 2:**
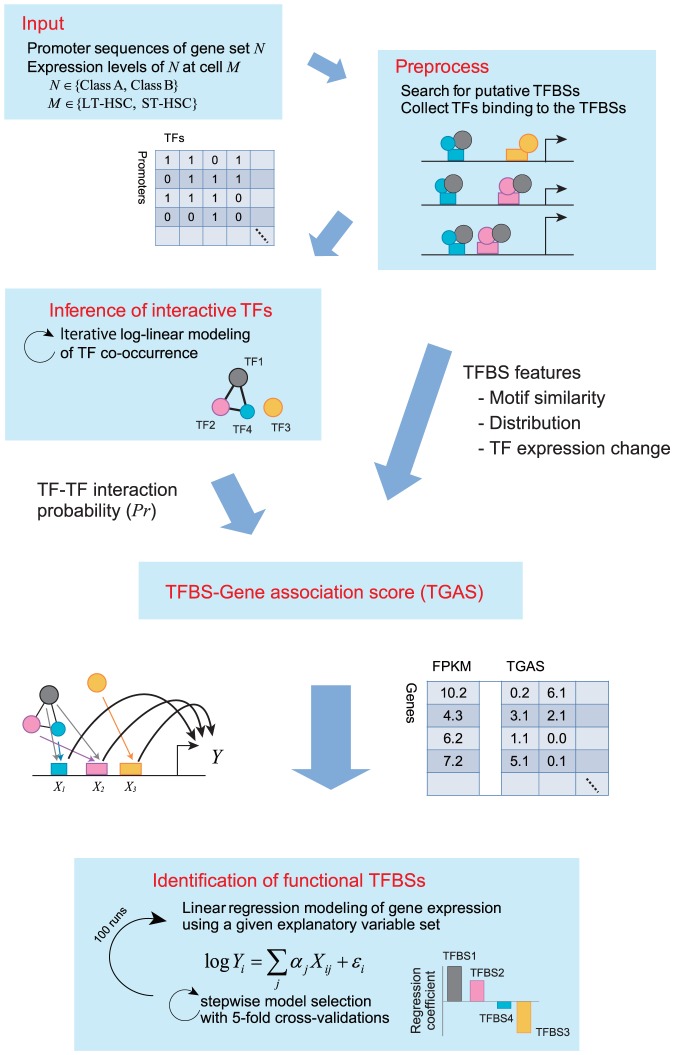
Overview of computational promoter modeling. We searched putative TFBSs and mouse TFs from 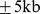 DNA sequences of TSSs, and used these for inferring TF–TF interaction probability and calculating TGASs. We searched the best combination of TFBSs represented by TGASs to predict FPKMs of a gene class in LT- or ST-HSCs by performing 5-fold CVs iteratively.

To identify regulators from proximal promoter regions comprehensively, we used TRANSFAC [39], which is a database that curates 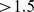 million ChIP-seq sites, and designed a workflow coupled with intensive computations (Figure 2). First, we prepared the promoter sequences of DEGs and searched for putative TFBSs and mouse TFs that are known to bind to the TFBSs in TRANSFAC using the MATCH tool [43]. This procedure identified 140 and 141 TFBSs for Class A and Class B promoters, respectively. Among these, 70 TFBSs in Class A and 69 TFBSs in Class B were targeted by at least one TF with a highly expressed coding gene (FPKM, 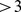). In total, 265 and 267 TFs were involved in Class A and Class B, respectively.

Next, we calculated the TFBS–gene association score (TGAS) using 5 distinct scoring schemes, which were employed as explanatory variables in a linear regression model. These scores considered matrix similarity, positional bias of TFBSs, the expression levels of TFs, and the probability of TF–TF interactions (Materials and Methods). Given a TGAS, we searched exhaustively for the best combination of TFBSs, including pairwise interactions between TFBSs. We performed a 5-fold cross-validation (CV) to avoid the risk of over-fitting. This procedure was repeated 100 times with different random seeds. An ensemble of 100 regression coefficients (RCs) for a TFBS provides statistical information of the estimated regulatory activity of the TFBS. We conducted statistical tests using these ensembles. We applied this workflow to 4 regression models to predict the expression levels of each of the Class A and Class B genes in LT- or ST-HSCs.

We attempted to characterize promoter architectures by testing the different TGASs mentioned above, rather than by comparing our approach with other modeling methods. This was because of the difficulty of implementing existing methods using our inputs and analyzing their results. We also aimed to determine regulatory activities by analyzing 4 models. We characterized the context-dependent function of regulators that activated and repressed the transcription of distinct genes depending on the cellular context [44], [45]. Thus, our approach provided a detailed picture of the regulatory modes involved in context-dependent gene expression.

#### Inference of higher-order TF interactions

The co-occupancy of a promoter by multiple TFs contributes synergistically to transcriptional regulation. We considered this when calculating TGAS by performing probabilistic LLM [31] coupled with iterative random sampling. The input matrix used for LLM, i.e., *n* promoters in rows 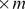 TFs in columns, comprised binary values that represent the existence of TFBSs for *m* TFs in *n* promoters. Using this matrix, LLM was employed to infer the conditional (in)dependency of TF occurrences, i.e., TF–TF interactions in higher-order conditional distributions. It should be noted that LLM cannot determine whether an interaction is competitive or cooperative.

The huge number of TFs means that LLM is not adequate to compute them all; therefore, we performed random sampling with 10 arbitrary selected TFs, which means that an inferred TF–TF interaction was observed constantly in the 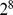 state combinations of 8 TFs. This sampling procedure was terminated if an outcome had no effect during 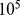 runs. We calculated the interaction probability *Pr* for all possible TF pairs using this iterative sampling procedure (Materials and Methods). After repeating the sampling procedure 1,367,639 times for Class A and 1,406,837 times for Class B, we retrieved 50 and 77 interactions (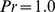) from Class A and Class B, respectively (Tables S12 and S13).

#### Performance of regression models

Overall, Pearson's correlation coefficient *R*s in learning and testing of 5-fold CVs showed a slight over-fitting in the range of 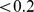 (Figure S5), which was acceptable in our sense. One of the reasons for this over-fitting was the unbalanced numbers between testing genes and TFBSs; e.g., 72 Class A genes (a subset of 5-fold CV) were tested by a model with over 100 predictors that were trained by the remaining Class A genes. This implies that the constructing of a model to generalize the gene regulation for an HSC population is a highly difficult challenge that is associated with the degrees of functional purity and heterogeneity and the limit of regulatory features used in the modeling.

Table 2 summarizes the results obtained from the linear regression models. The results showed that TGAS V coupled with LLM had the highest mean *R* between the observed and predicted gene expression. Interestingly, TGAS IV, which removed TFBSs where all TFs had FPKM 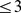, yielded poor–quality models, suggesting that these TFBSs were also necessary for modeling gene regulation. In addition, compared with the main effect terms (denoted as ''single'' in Table 2), a large number of pairwise terms, i.e., 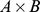, where *A* and *B* are 2 distinct TFBSs that were not included as main effect terms, contributed to the modeling. Indeed, the initial models that comprised only the main effect terms selected on the basis of Akaike's information criterion (AIC) showed an *R* 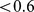.

**Table 2:** Result obtained using the linear regression models.

			Linear regression		TFBS contents	
Class	Cell	TGAS*	TFBSs	R†	Single	Pairwise
A	LT-HSC	I	83.91 (5.7238)§	0.8016 (0.0205)	18.98 (2.1070)	64.93 (5.7745)
		II	98.69 (5.5492)	0.8482 (0.0197)	30.67 (2.6685)	68.02 (5.8378)
		III	103.73 (4.5296)	0.8722 (0.0134)	29.14 (2.5220)	74.59 (5.0062)
		IV	47.28 (2.9260)	0.6771 (0.0165)	11.15 (1.2835)	36.13 (2.9888)
		V	108.12 (4.9138)	0.8850 (0.0124)	31.82 (2.6921)	76.30 (5.1449)
		V-1	84.42 (4.0748)	0.8334 (0.0154)	18.59 (6.3594)	65.83 (7.0129)
		V-2	51.90 (2.9648)	0.7164 (0.0155)	11.96 (1.1128)	39.94 (3.0588)
		V-3	91.38 (4.3053)	0.8284 (0.0146)	28.01 (2.5120)	63.37 (4.8634)
	ST-HSC	I	83.02 (5.3907)	0.8087 (0.0204)	20.66 (1.9709)	62.36 (5.9389)
		II	101.65 (4.7188)	0.8463 (0.0180)	37.47 (2.8088)	64.18 (5.6416)
		III	106.77 (4.0394)	0.8730 (0.0114)	36.29 (2.7579)	70.48 (4.5902)
		IV	50.34 (3.1376)	0.6786 (0.0215)	17.63 (2.0768)	32.71 (3.6064)
		V	108.49 (4.5618)	0.8777 (0.0132)	37.62 (2.7378)	70.87 (5.2548)
		V-1	85.01 (4.2883)	0.8289 (0.0160)	22.75 (2.1372)	62.26 (4.3327)
		V-2	53.32 (3.1012)	0.6867 (0.0191)	21.53 (2.2470)	31.79 (3.5222)
		V-3	86.71 (4.8853)	0.8126 (0.0196)	26.03 (2.6399)	60.68 (5.4934)
B	LT-HSC	I	77.82 (5.6451)	0.6177 (0.0183)	21.98 (2.1400)	55.84 (6.2749)
		II	100.86 (4.3244)	0.7016 (0.0147)	30.33 (2.8002)	70.53 (4.9748)
		III	105.78 (3.8251)	0.7311 (0.0125)	27.96 (2.4614)	77.82 (4.1434)
		IV	49.50 (2.8231)	0.5410 (0.0143)	15.29 (1.7164)	34.21 (3.1058)
		V	108.45 (4.2270)	0.7466 (0.0111)	27.20 (2.4819)	81.25 (4.3183)
		V-1	87.86 (3.7895)	0.6736 (0.0159)	28.59 (3.1051)	59.27 (4.9272)
		V-2	53.74 (2.7879)	0.5548 (0.0145)	15.54 (1.5324)	38.20 (3.1969)
		V-3	84.45 (3.8350)	0.6662 (0.0149)	24.95 (2.5744)	59.50 (4.6573)
	ST-HSC	I	77.65 (4.7924)	0.6077 (0.0175)	21.42 (2.0745)	56.23 (5.3514)
		II	100.69 (5.3846)	0.6980 (0.0169)	25.74 (2.4602)	74.95 (6.0056)
		III	105.87 (4.1633)	0.7262 (0.0140)	24.73 (2.4448)	81.14 (4.8477)
		IV	50.07 (2.8679)	0.5160 (0.0161)	14.36 (1.7235)	35.71 (3.3920)
		V	107.32 (4.4763)	0.7325 (0.0135)	24.77 (2.6338)	82.55 (4.6720)
		V-1	86.98 (3.8781)	0.6716 (0.0166)	22.62 (2.4891)	64.36 (4.6206)
		V-2	54.09 (2.9397)	0.5354 (0.0164)	15.65 (1.8993)	38.44 (3.5080)
		V-3	84.96 (4.1639)	0.6544 (0.0165)	21.47 (2.4185)	63.49 (4.9830)

E.g., at the top line, the final regression model predicted Class A FPKMs in LT-HSCs using TGAS I, resulting in the correlation coefficient 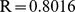. This model included 83.91 TFBSs consisting of 18.98 single TFBSs and 64.93 pairwise TFBSs.

**Figure 3:**
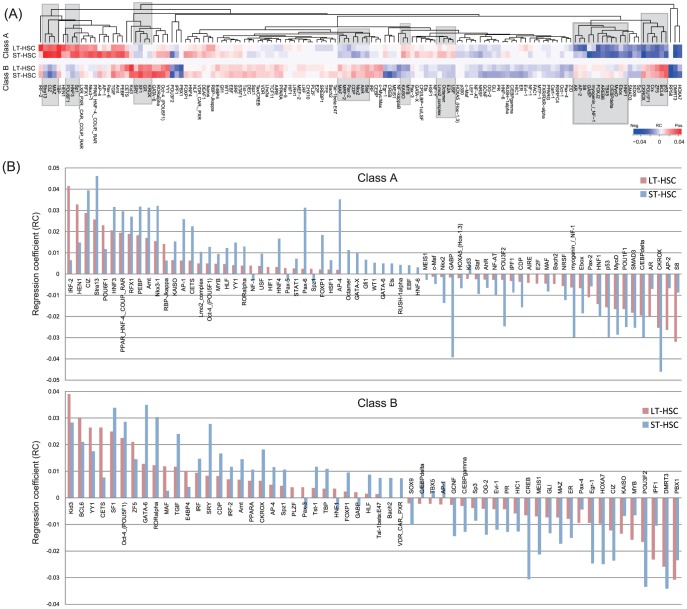
Propensity of significant TFBS activities. (A) Heat map showing regression coefficients (RCs) of 142 potentially important TFBSs (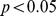). Rectangles indicate typical cases of opposing RCs in Class A and Class B. (B) We found that 71 and 58 TFBSs from Class A and Class B promoters, respectively, had significantly different RCs in LT- and ST-HSCs (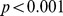).

**Figure 4:**
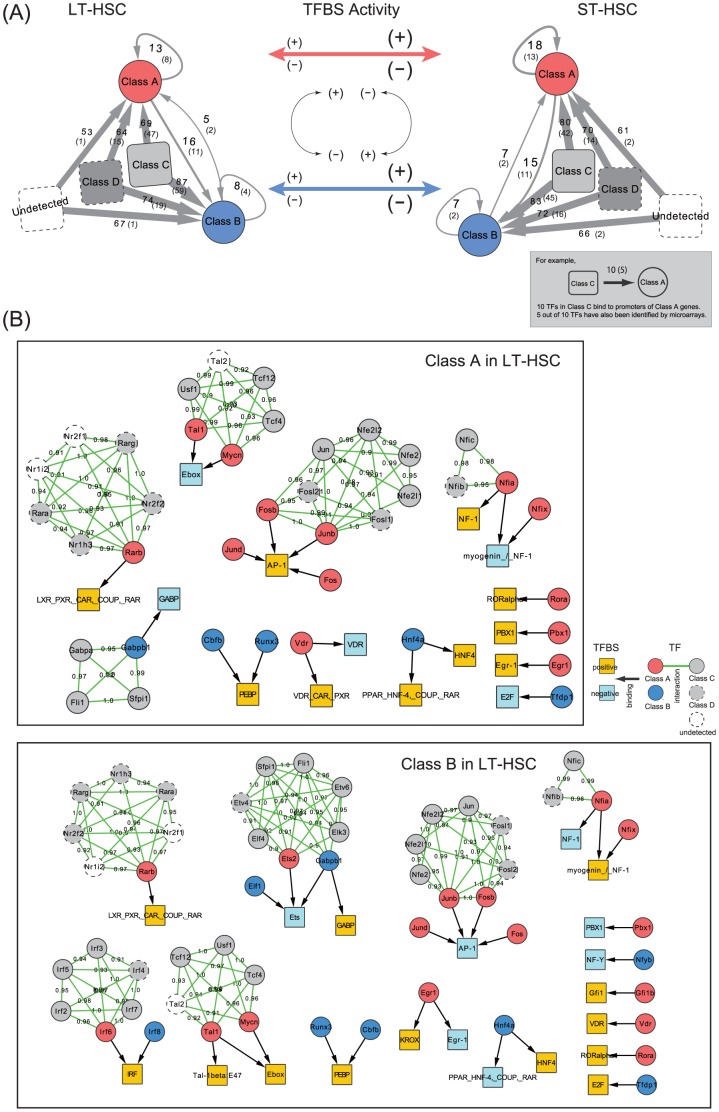
Inference of transcriptional regulatory networks. (A) Systematic representation of TF–gene networks and the change of TFBS activities between LT-HSCs and ST-HSCs. Genes that produce TFs that putatively bind to important TFBSs (Figure 3A) existed in each class. Some of them were not detected in the RNA-seq assay, and were categorized as ''Undetected''. The numbers on the gray-colored arrows denote the number of TFs in the corresponding class that bind to Class A or B gene promoters, suggesting that the majority of TFs belonged to nondifferentially expressed gene classes. The numbers in parentheses indicate TFs that were detected in microarray-based studies, suggesting the extensive discovery of our assay. As shown in the middle panel, we inferred that the positive or negative activities of TFBSs are mostly unchanged between cells, but are inverted between Class A and Class B. (B) Subnetworks of (A) in LT-HSCs. The majority of TF-coding genes were not differentially expressed, whereas 24 TFs binding to 21 TFBSs were present among DEGs (Class A and Class B) and interacted strongly with nondifferentially expressed TFs (Figure S4 shows the subnetworks in ST-HSCs).

*TFBS-Gene association scores; (I) MATCH score only, (II) including distribution of TFBSs, (III) including expression changes in TFs, (IV) same as (III) but only including TFBSs targeted by highly expressed TFs, and (V) including the TF–TF interactions in the log-linear model. (V) was modified to remove TFs: coded by undetectable transcripts (V-1), those that belonged to Class D (V-2), or by removing the 21 TFBSs in Figure 4B (V-3).

† Pearson's correlation coefficient; once the final regression model was found, *R* reflecting the model quality is calculated to measure the degree of correlation between the observed and predicted FPKMs.

§ Data are presented as the means (and standard deviation in parentheses).

The improvement observed using TGAS V compared with the use of TGAS III was not remarkable. To assess this improvement, we performed a two-sample *t*-test using RC ensembles of TFBSs that were common in the 2 models. This analysis indicated that these models yielded considerably different TFBS activities (Figure S2A). In most cases, TF interaction scores (Equation 9) were ineffectively small. However, specific TFBSs, such as AP-1, Ets, and Ebox, had high scores (Figure S2B) because of the relatively larger number of TFs that interacted to occupy these TFBSs (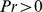). This apparently affected the different estimations.

Overall, pairwise interactions between TFBSs reflected regulatory modules that appeared to be essential components of the transcriptional machinery. The incorporation of cooperative and competitive interactions among TFs into quantitative models is also essential for determining the transcriptional network based on a fine-tuned explanation of gene expression.

### Propensity of inferred TFBS activities

#### Identification of significant TFBSs and changes of regulatory activities

To assess the statistical significance of TFBS activities, we performed single-sample *t*-tests using RC ensembles on the basis of TGAS V (Figure S3). This analysis identified 142 TFBSs that rejected the null hypothesis that the mean value of RCs was equal to zero (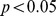). This included several known hematopoietic regulators, such as Arnt, C/EBP, CREB, Ebox, Egr-1, GATA-X, and IRF (Figure 3A). In particular, GATA-X targeted by *Gata1*, *Gata2*, or *Gata3* (Class A) was significant only in the model of Class A in ST-HSCs. Consistent with a recent analysis of *Hlf* function [25], we inferred positive RCs for HLF in all 4 models, which suggests that it functions as an activator. *Hlf* was upregulated by 1.9-fold in LT-HSCs (Class C). We also validated PPARG activity using a competitive repopulating assay (see below).

Overall, 83 of the 142 TFBSs were detected by all 4 models, among which 14 TFBSs were unique in Class A or Class B (Table S6). Furthermore, 73% (61/83) of the common TFBSs appeared to have same effects on the activities in LT- and ST-HSCs, e.g., a positive RC in LT-HSC was also positive in ST-HSC. Interestingly, this effect was the opposite in Class A and Class B, for which typical examples are marked by rectangles in Figure 3A. There were also exceptional cases, including IRF-2, HOXA7, and DMRT3. The results obtained using TGAS III had similar properties.

#### Gain and loss of activities during HSC progression

To analyze the change of TFBS activities between LT- and ST-HSCs, we tested 2 RC ensembles of a TFBS using a two-sample *t*-test under the null hypothesis that the mean values were equal. This analysis found that the null hypothesis was rejected for 71 TFBSs (Class A) and 58 TFBSs (Class B) (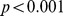) (Figure 3B). The multiple-testing correction reduced these numbers to 49 and 42 in Class A and Class B, respectively (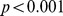) (Tables S8 and S9). Interestingly, although these TFBSs had different mean values, the effects of the activities were mostly unchanged; a positive (negative) activity in LT-HSC was still positive (negative) in ST-HSC, i.e., 75% (53/71) in Class A and 84% (49/58) in Class B. In most cases, the strengths of these activities increased markedly in ST-HSC, i.e., 85% (45/53) in Class A and 76% (37/49) in Class B. These results suggest that the maintenance of self-renewal and the differentiation competence in ST-HSCs require a vigorous transcriptional program.

As an intuitive insight into the gain and loss of activities during HSC progression, we found that downregulation of Class A in ST-HSC relative to LT-HSC was accompanied by a gain of negative RCs in ST-HSC (e.g., CKROX, GABP, C/EBPdelta, and myogenin/NF-1) and by a loss of positive RCs in LT-HSC (e.g., IRF-2, HEN1, POU6F1, and RBP-jkappa). Similarly, upregulation of Class B in ST-HSC relative to LT-HSC was followed by a gain of positive RCs in ST-HSC (e.g., SF1, Oct-4, GATA-6, and RORalpha) and by a loss of negative RCs in LT-HSC (e.g., PBX1, IPF1, MYB, KAISO, and Pax-4). However, many of the TFBSs in each class exhibited activity changes that differed greatly from our intuitive expectations, which suggests that the high level of complexity in the transcriptional circuit is related to context-dependent gene expression.

### Functional importance of TFs coded by rare transcripts

#### Regulatory effects of TFs from gene classes that were expressed at low and undetectable levels

We constructed TF–gene networks on the basis of the links between the 142 TFBSs and their downstream target genes. The networks had vast numbers of edges: 40,896 edges among 204 TFs that targeted 114 TFBSs of Class A in LT-HSC; 45,882 edges among 237 TFs that targeted 114 TFBSs of Class A in ST-HSC; 97,946 edges among 253 TFs that targeted 134 TFBSs of Class B in LT-HSC; and 96,975 edges among 243 TFs that targeted 125 TFBSs of Class B in ST-HSC.

The majority of TFs involved in these networks belonged to Class D and transcripts that were not detected in our RNA-seq assay (Figure 4A). Only a small portion of these genes were detected by microarray analyses [8], [9], i.e., the numbers in parentheses in Figure 4A. Our qRT-PCR assay detected only 1 or 2 of these genes, suggesting that they originated from rare transcripts, i.e., TF-coding genes expressed at low or undetectable levels in HSCs. To assess the importance of these TFs, we modified TGAS V to remove the regulatory effects from the TFs; by setting 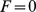 (Equation 8) for unexpressed TF-coding genes (TGAS V-1) and for TF-coding genes in Class D (TGAS V-2). As a result, *R*s were lower than TGAS V when the model removed these effects (Table 2), which suggests their important contribution to the modeling. Indeed, many known factors [17], [46] were present in these categories.

#### Competitive repopulation assay with activated *Pparg*

The suggestion that TF-coding genes expressed at low levels are important contributors to transcriptional regulation prompted us to investigate the function of *Pparg*, which remains controversial in HSC biology [47]. *Pparg* was categorized into Class D (0.3747 FPKM in LT-HSC and 0.2616 FPKM in ST-HSC), and its binding site PPARG had negative RCs in all 4 models (Figure 3A). To confirm this PPARG activity, we treated LT-HSCs with GW1929, a high agonist of *Pparg* [48], [49].

**Figure 5:**
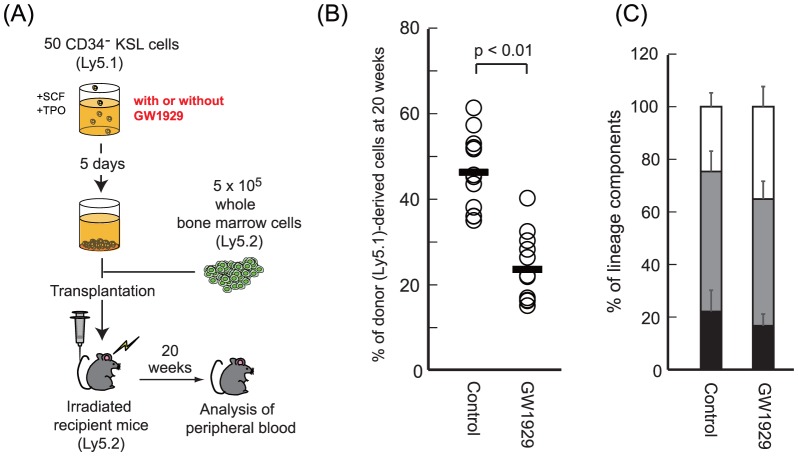
Long-term competitive reconstitution assay. (A) Scheme of the competitive repopulation assay using GW1929, a high agonist of *Pparg*. (B) Analysis of the proportion of donor-derived 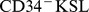 HSCs obtained from untreated (Control) and treated (GW1929) recipient mice at 20 weeks posttransplant. Each dot represents a single mouse. (C) Relative contributions of 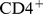 or 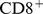 (T-cell lineage), 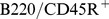 (B-cell lineage) and Mac-1^+^ or Gr-1^+^ (myeloid lineage) cells in donor-derived Ly-5.1^+^ cells of recipient mice at 20 weeks posttransplant. Black, T-cell lineage; gray, B-cell lineage; white, myeloid lineage. Data are presented as the mean 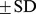.

As shown in Figure 5A, we performed a transplantation assay using LT-HSCs that were cultured for 5 days with or without GW1929. GW1929-treated HSCs exhibited decreased chimerism at 20 weeks after the transplantation compared with the controls (Figure 5B). The contribution of T-cell, B-cell, and myeloid lineages to the total donor-derived cells was not highly different (Figure 5C). These results suggest the possibility that the excessive activity of PPARG influences negatively the long-term repopulating activity of HSCs, which supports the capacity of our approach to infer the activities of regulatory elements in HSCs.

### Identification of potential key regulators

#### Differentially expressed TFs and their target sites

The regulatory networks (Figure 4A) involved differentially expressed TFs, i.e., 18 TFs regulated Class A in LT-HSC (13 from Class A and 5 from Class B) and 24 TFs regulated Class B in LT-HSC (16 from Class A and 8 from Class B). These TFs targeted 21 TFBSs that are well-studied hematopoietic regulators, including the Fos/Jun complex [50], Ebox-binding bHLH TFs [51], the GABP complex [52], and retinoic acid receptors [53]. In particular, AP-1 and Egr-1 appeared in all of the models and were targeted by immediate early response genes that are important for apoptosis and differentiation [50] and that are downregulated in ST-HSCs [54]. Interestingly, our model showed that some of these TFs are highly modulated by other TFs that were not differentially expressed (Figures 4B and S4). This may explain the observation that the models with TGAS V-1 and TGAS V-2 reduced the predictive performance.

#### Putative function of the differentially expressed TFs

**Figure 6:**
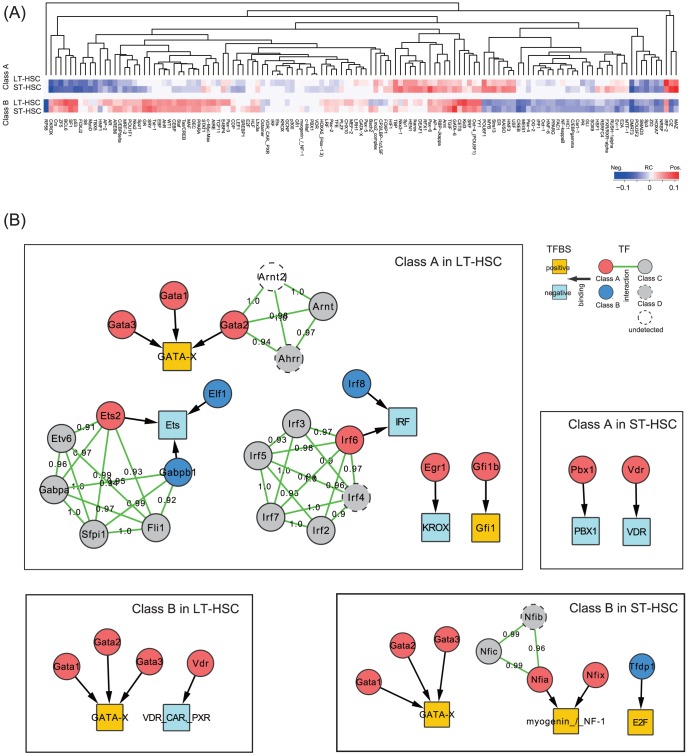
Alternative regulators potentially important in the presence of dysfunctional TFBSs that are targeted by differentially expressed TFs. (A) Heat map showing the regression coefficients (RCs) of 129 potentially important TFBSs (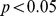) that were identified after the removal of the TFBSs in Figures 4B and S4. The overall propensity of TFBS activities were not different from those shown in Figure 3A. (B) This removal test identified subnetworks that involve alternative TFBSs targeted by differentially expressed TFs. These included GATA-X, Ets, and IRF, which are related to erythroid/megakaryocytic lineage commitment; 6 TFBSs were targeted by 11 TFs in LT-HSCs, and 5 TFBSs were targeted by 8 TFs in ST-HSCs.

Many recent studies have reported that epigenetic effects are important factors in hematopoiesis [16], [18], [55]. What would happen if the 21 TFBSs targeted by differentially expressed TFs were turned off by DNA methylation, for example? This question was suggested by the recent finding that CpG-methylated regions colocalize with TFBSs in HSCs [56]. To answer this question, we removed each set of TFBSs that appeared in Figures 4B and S4, and performed regression modeling in this condition. The results showed slightly lower *R*s (TGAS V-3 in Table 2), however, the overall propensity of the activities was not different from those shown in Figure 3A (Figure 6A).

Interestingly, specific TFBSs (e.g., GATA-X, Ets, and IRF) that were targeted by differentially expressed TFs were determined (Figure 6B). The most remarkable change was that GATA-X acquired positive activities in LT-HSCs. It is well known that GATA and AP-1 frequently co-occupy chromatin sites and that they play critical roles in cell fate decisions to commit to erythroid vs. myeloid lineages [57], [58]. More recent studies have shown that epigenetic marks control the interactions among Gata factors and other hematopoietic TFs [55], and that the DNA methylation patterns of the GATA and AP-1 motifs are mutually exclusive during early hematopoiesis [56].

Overall, our results suggest that the 24 TFs that target 21 TFBSs (Figure 4B) are key regulators of HSCs. The ST-HSCs used here exhibited lymphoid-priming features [8] with preferentially repressive potential megakaryocyte/erythroid genes (Table S7). Therefore, these regulators may be related to lymphoid-lineage development. Our model showed that dysfunctions in these regulators led to alternative regulators related to erythroid/megakaryocytic lineage development competence. This supports the recent remarkable finding of a novel lineage commitment pathway [4].

## Discussion

HSC fate is controlled tightly by extrinsic and intrinsic factors [1], [2], [10]-[12], [36]. The identification and characterization of these factors may lead to more effective clinical therapies for acquired and congenital blood disorders. Owing to recent advances in experimental and computational techniques, many recent studies [3], [4], [25] have begun to move beyond the traditional beliefs regarding hematopoiesis. However, the determination of the upstream regulatory elements that are responsible for the development of the hematopoietic system remains far from adequate and requires the application of various approaches. In the present study, we established novel transcriptome profiles from mouse LT- and ST-HSCs using an RNA-seq assay and developed a computational method for exploring the potential modes of transcriptional regulation based on these profiles.

Our RNA-seq assay confirmed the transcriptionally active state of ST-HSCs [6], [7], [15] with markedly high numbers of DEGs. These DEGs included 77 cell-surface molecules and 57 TFs (Tables 1 and S2–S5), which indicates that specific extrinsic and intrinsic regulators respond actively during the transition between LT- and ST-HSCs. During this transition, we observed that many previously annotated lineage-specific genes [8] were up- and downregulated (Table S7). In particular, lymphoid potential genes that preferentially undergo upregulation in ST-HSCs and potential megakaryocyte/erythroid genes had opposite patterns, suggesting that lymphoid priming occurs during this early stage.

To investigate the regulatory activities of known factors, we conducted a preliminary study using our previous method [29] and ChIP-seq data for 10 major hematopoietic regulators [17]; however, we were unable to obtain any significant results (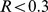). This failure prompted us to extend our approach in the following manner (Figure 2). To approximate TFBS activities, we employed cis- and trans-regulatory information from TRANSFAC [39]. Furthermore, to consider the combinatorial regulation of TFs, we incorporated the probabilities of the conditional TF–TF interactions inferred by LLM [31]. Thus, our approach systematically inferred the regulatory activities of TFBSs, and suggested potential synergistic TF modules. Consequently, we found that motif similarity, the positional distribution of motifs, and expression changes in TFs were the most informative features for the promoter modeling of DEGs. Using LLM, we quantified the TFBS activities on the basis of the fine-tuned explanations of DEGs (TGAS V in Table 2).

Many hematopoietic TFs [6], [17] were included among the transcriptional steady-state gene set (Class C), the low-level expression gene set (Class D), or the genes expressed at undetectable levels. Throughout this study, we found that the regulatory effects of these TFs and their target sites are essential to explain the regulation of DEGs. This may explain, in part, the observation that our preliminary model using 10 major hematopoietic TFs was not well fitted. We further supported this finding by performing a transplantation assay of LT-HSCs cultured with activated *Pparg* (Figure 5). Furthermore, we found that these TFs modulated differentially expressed TFs that are likely to be important during commitment to specific lineages (Figures 4B and 6B). However, LLM inferred low probabilities for interactions between known co-operative TF pairs (Tables S12 and S13), e.g., *Gata2* and *Erg* (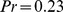 in Classes A and B) and *Gata2* and *Tal1* (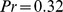 in Class A, 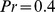 in Class B), which suggests that their co-operation regulates specific gene sets.

We identified 142 TFBSs that contributed significantly to the regression models (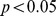). Among these, 71 TFBSs (Class A) and 58 TFBSs (Class B) exhibited a considerable gain or loss of their activities during cell differentiation (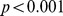). As illustrated in Figure 4A, the effects of TFBS activities represented by plus or minus signs of RCs were mostly unchanged between cells but were inverted between DEGs. The strengths of TFBS activities increased markedly in ST-HSCs compared with LT-HSCs. We applied our method to 2 public RNA-seq datasets that represented sequential cell development (MII oocytes and two-cell embryos) and lineage commitment (megakaryocyte/erythroid precursors and megakaryocytes) (Figure S5). This analysis showed that the results of cell-lineage commitment agreed with the propensity of the regulatory activities detected in HSCs, rather than with that of sequential cell development. Therefore, regulators that play similar or different roles in accordance with cellular contexts might be general features that underlie cell fate decisions.

Overall, our results suggest that HSCs exhibit flexible and rapid responses to local needs by controlling TFs that are expressed at steady-state or low levels via a highly complex regulatory network. Further studies should consider the implications of these regulatory modes based on instructive and/or stochastic models of stem cell fate decisions. In the present study, we demonstrated that specific lineage-affiliated TFs formed a resultant set of transcriptional regulation, i.e., 24 differentially expressed TFs that contributed significantly to the model were modulated by other TFs that were not differentially expressed. These TFs include immediate early genes (e.g., *Fos*, *Jun*, and *Egr1*) that induce an early genomic response related to HSC biology [50], [54]. If they become dysfunctional, LT-HSCs may be primed to an erythroid/megakaryocytic lineage via pathways that are controlled by other TFs (e.g., Gata factors, ETS family, and IRF family).

In summary, we obtained novel transcriptome data and developed a computational method for promoter modeling. Our method can be applied easily to other biological systems. Using these approaches, we identified transcriptional regulation modes that provide insights into how HSCs determine their phenotype. Future works that overcome the limitations of the present study, such as the inclusion of enhancer activities that appear to be important in hematopoiesis [17], [42] and the analysis of the influence of transcriptional heterogeneity at the single-cell level [4], [10], [34], which can be assayed using promising techniques [59]–[61], would refine our findings and advance our understanding of the kinetic and regulatory aspects of stem cell biology.

## Materials and Methods

### Animals

All experimental protocols were reviewed and approved by the Institutional Animal Care and Use Committee of Tokyo Women's Medical University (approval ID: 13-99-2-B). Mice were purchased from Sankyo Labo Service.

### Cell collection


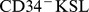
 (c-kit^+^Sca1^+^Lin^−^) LT-HSCs or 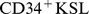 ST-HSCs were sorted, as described previously [36]. In brief, we isolated bone marrow cells from 8- to 10-week-old C57BL/6 mice and stained them with antibodies for CD34 (RAM34, eBiosciences, San Diego, CA), Sca-1 (E13-161.7, BD Biosciences Pharmingen, San Jose, CA), c-kit (2B8, BD Biosciences Pharmingen), and a lineage marker (Lineage Detection Kit, Miltenyi Biotec Inc., Bergisch Gladbach, Germany). Subsequently, we analyzed the stained cells using a MoFlo XDP cell sorter system (Beckman Coulter, Fullerton, CA).

### RNA sequencing and real-time PCR

After obtaining total RNA extracts from 5000 LT- or ST-HSCs using Isogen (Nippon Gene, Tokyo, Japan) in triplicate, we synthesized cDNA using a SMARTer Pico cDNA amplification kit (Clonetech, Mountain View, CA) and amplified them with 20 cycles of PCR. Using the standard protocols for the SOLiD system, we sequenced the amplified cDNA using a SOLiD sequencer (Life Technologies, Carlsbad, CA), as described previously [36]. In the RT-PCR assay, total RNA was obtained from the sorted cells and cDNA was synthesized as described above. We performed RT-PCR using a TaqMan Gene Expression Assay (Life Technologies) for the genes indicated with the BioMark HD system (Fludigm, South San Francisco, CA).

### Read mapping and quantification

We used the TopHat (v1.4.1)/Cufflinks (v.2.0.2) pipeline [33] with the sequenced reads (quality score, 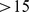). The pipeline was coupled to Bowtie (v.0.12.7) [62]. We employed the recursive read mapping method, as described previously [32]. In brief, we applied TopHat by truncating the 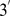 ends of unmapped reads and by realigning the reads using more stringent parameters. We set the parameters empirically, which were used sequentially, as the read length, ''-initial-read-mismatches'', ''-segment-mismatches'', and ''-segment-length'': (50, 3, 2, 25), (46, 3, 2, 23), (42, 3, 2, 21), (38, 2, 0, 19), and (34, 2, 0, 17).

The pipeline, which quantifies RNA abundance as fragments per kilobase of exon per million mapped reads (FPKM), mapped sequenced reads to the mouse genome (mm9), and then assembled transcripts with uniquely mapped reads (uni-reads) for each replicate. We used Cuffcompare to merge all the transcript assemblies; 14,728 and 14,128 RefSeq-annotated genes in LT- and ST-HSCs, respectively. Using the merged transcript assembly, we performed Cuffdiff, which calculates FPKMs across all replicates and detects DEGs via two-group *t*-tests coupled to a Benjamini–Hochberg false discovery rate (FDR) procedure. We further used transcripts that satisfied the following conditions: successful deconvolution, FDR of 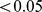, complete match of intron chain, and FPKM of 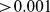. The mouse genome and RefSeq annotation were downloaded from http://genome.ucsc.edu/.

### Long-term competitive reconstitution assay

We cultured 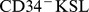 HSCs derived from C57BL/6-Ly5.1 congenic mice for 5 days with or without 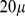
M GW1929 (Sigma-Aldrich, St. Louis, MO) in S-Clone SF-03 medium (Sanko-Junyaku Co., Tokyo, Japan) supplemented with 0.5% bovine serum albumin (Sigma, St. Louis, MO) and 50 ng/ml mouse stem cell factor and 50 ng/ml mouse TPO (all from R&D systems, Minneapolis, MN). Next, we performed a long-term competitive reconstitution assay by transplanting cultured cells with 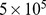 whole bone marrow competitor cells derived from C57BL/6-Ly5.2 Wt mice into lethally irradiated (9.5 Gy) C57BL/6-Ly5.2 Wt mice.

### Log-linear model (LLM)

Suppose that we consider binary-stated (absence or presence) TFs {*A, B, C*}. The observed counts fall into 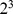-dimensional contingency table by cross-classifying the TF states. The full model (FM), which contains all the possible interactions, gives the logarithms of probabilities as follows:
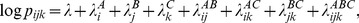
(1)where *i, j* and *k* are the state indices of {*A, B, C*}, 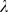
s are unknown parameters, 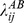, 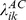 and 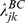 represent the interaction effects among the indexed variables. If an instance of *A* is independent of *B*, FM can be reduced to a reduced model (RM) with respect to the hierarchy [31], which is given as follows:
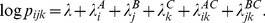
(2)


This model can be reformulated as
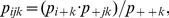
(3)where ''+'' denotes the summation over the corresponding index. This formula is equivalent to 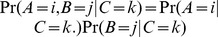, which means that *A* and *B* are independent in the conditional distribution given *C* (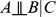).

To find the most parsimonious RM, we remove an interaction term from the current model and measure two *p*-values for the asymptotic 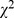 test of a likelihood ratio 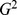 statistic [31]. The *p*-values comprise *p*_FM, which is the difference between FM and RM, and *p*_RM, which is the difference between the current model and RM. We accept a removal if it yields the largest *p*_RM (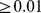), and we terminate if any removal test yields 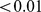 for either *p*_RM or *p*_FM.

### Iterative random sampling for LLM

A large number of TFs can easily yield a vast dimensional contingency table. To find a near optimal parsimonious model even in such higher-dimensional space, we designed an iterative sampling scheme that allowed us to calculate interaction probability *Pr* as follows.

Let 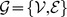 is an undirected graph, where 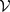 is a finite set of vertices (TFs) and 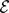 is a set of edges, which represent the interactions between vertex pairs. The scheme is as follows.

1. 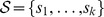, a nonredundant combination of TFs, is selected randomly from all TFs (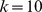 in the present study).
For all possible vertex pairs 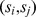, the trial number 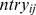 of an edge between 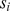 and 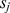 is counted (i.e., FM of *k* variables).
LLM infers the best model 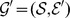, where 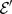 is a set of edges that represents TF–TF interactions.
For all possible vertex pairs 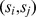, if an edge in 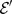 links a pair 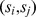, the observed edge frequency 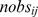 for this pair is counted.
For all possible vertex pairs 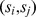, the interaction probability *Pr* for a pair 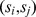 is updated using 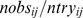.
If 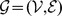, where 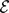 is a set of edges (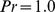), is not changed with a large number of samplings (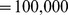); therefore, this procedure is terminated. Otherwise, steps 1–5 are repeated.

### Linear regression model

We used a multivariate regression model
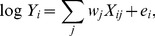
(4)

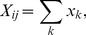
(5)where 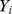 is the expression of gene *i*, 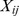 is TGAS of the *j*th TFBS in the promoter region of gene *i*, 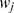 is RC of the *j*th TFBS, and 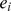 is the error term. TGAS is the sum of scores 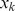, where *k* represents the position of the *j*th TFBS in promoter *i*. We tested the following forms of 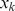.

- I: matrix similarity *s* of TFBS *j* scored using MATCH [43] (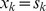).
- II: TGAS I modified by a location-dependent weight *L*,
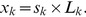
(6)
- III: TGAS II weighted by the expression fold change (*F*) of TFs,
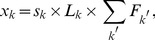
(7)where 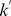 is the index of TFs binding to TFBS *j*. If FPKM for TF is 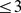, we use 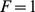.
- IV: the same as TGAS III, but we removed TFBSs where none of the TFs had FPKM of 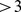.
- V: TGAS III weighted using both *F*s of interactive TFs and the interaction probability *Pr* estimated by LLM,
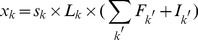
(8)

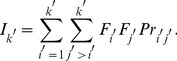
(9)

We used a published method to calculate *L* [40]. First, we calculated the distribution of TFBS *j* in bins ( = 500 bp) of promoter regions and created a histogram 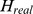. Next, we randomized the positions of TFBS *j* and created a histogram 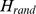. *L* for the *k*th TFBS *j* is given by the following:
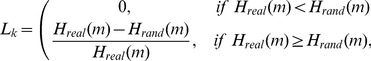
(10)where *m* represents the index of bin that corresponds to the position of the *k*th TFBS *j*. This location-dependent weight takes a value between 0 and 1, where a higher weight implies nonrandom occurrence.

### Stepwise selection of the regression model

We built a regression model with the explanatory variable *X* and then reduced the model using AIC. Let the reduced model be 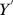 with 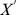. 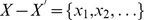 is the variables removed on the basis of AIC. *V* is the set of all pairwise terms of 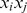 (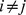). We searched any elements of *V* that improve Pearson's correlation coefficient 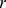 of 5-fold CV on testing datasets.

1. Randomly select 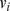 (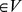) and add it to 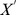, which yields 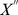.
2. Perform 5-fold CV with 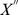 and calculate the averaged *r* on testing datasets.
3. If the *r* has been improved, update 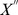 to 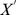.
4. Repeat step 1–3 until all 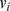 have been tested.
5. Calculate Pearson's correlation coefficient *R* between observed and predicted FPKMs of all genes by using the final model.

We run this procedure 100 times using different random seeds. The final *R* is referred to as a model quality in this study.

### Bioinformatics analysis

We obtained array-based gene expression profiles [8], [9] from BloodExpress [63], RNA-seq data for megakaryocyte/erythroid precursors and megakaryocytes from http://genome.ucsc.edu/encode/, and RNA-seq data for MII oocytes and two-cell embryos from DDBJ DRA001066. The public RNA-seq datasets were analyzed using the pipeline mentioned above. To search putative TFBSs and TFs in TRANSFAC professional (released in January 2013) [39], we prepared 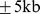 DNA sequences from transcription start sites (TSSs) annotated in RefSeq (http://www.ncbi.nlm.nih.gov/refseq/), and applied the MATCH tool in the minimize false-positive mode [43].

To analyze the enriched GO terms, we used the DAVID Bioinformatics Resources [35]. Significant terms detected by DAVID (EASE score, a modified Fisher's exact *p*-value, 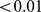) were grouped into representative ancestor terms in the dataset GO Slim2 using CateGOrizer [64]. We used the R programming language (http://www.r-project.org/) for regression modeling and to perform statistical tests. Although all *p*-values were adjusted by Bonferroni correction (Tables S6 and S8–S11), we used uncorrected *p*-values throughout this study to avoid too conservative interpretation that would reduce biologically meaningful findings.

### Data access

The RNA-seq data generated in this study have been deposited in the DDBJ (DNA Data Bank of Japan) Sequence Read Archive (DRA) under accession number DRA001213. The online version of LLM is available at http://dbtmee.hgc.jp/tools/.

## Supporting Information

Figure S1
**Correlation analysis of gene expression levels measured using RNA-seq assays.** (A) Reproducibility based on triplicate analyses of LT- and ST-HSCs. (B) Comparison of the gene expression correlations in the present study to those reported by Karlsson et al. [15], who purified HSCs using CD48^−^, CD150^+^, CD34^−^, CD9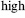 KSL for LT-HSCs and CD48^−^, CD150^+^, CD9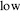 KSL for ST-HSCs.(EPS)

Figure S2
**Contribution of higher-order TF interaction scores estimated by LLM.** (A) Statistical differences of 2 regression coefficient (RC) ensembles of a TFBS found commonly by TGAS III and V (two-sample *t*-test). (B) Distribution of the TF interaction score 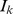 in Equation 9.(EPS)

Figure S3 **Box plots of RCs estimated by 100 iterations of regression modeling with TGAS V.** Pos and Neg represent the positive (red) and negative (blue) mean values of RCs (red line), respectively.
(EPS)

Figure S4 **Subnetworks involved in ST-HSC regulation.** Although the majority of TF-coding genes found in ST-HSCs (Figure 4A) were not differentially expressed, 26 differentially expressed TFs that putatively bind to 21 TFBSs were present among DEGs (Class A and Class B).
(EPS)

Figure S5
**Propensity of the TFBS activities inferred from public RNA-seq datasets.** We applied our method to public RNA-seq datasets related to sequential cell development (A) and lineage commitment (C). Our procedure evaluates the averaged *R* of 5-fold CV on testing datasets (blue line). If a model improved *R* in testing, the model was accepted and its *R* value between the observed and predicted gene expression of all genes was measured (red line). (B) Of 147 TFBSs (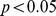), 67 TFBSs (Class A; upregulated in Oo) and 80 TFBSs (Class B; upregulated in 2C) exhibited significant gains and losses of activity (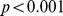). In addition, 73% (49/67) of Class A and 52.5% (42/80) of Class B genes exhibited no changes in the effects of their TFBS activities between cells, i.e., positive (negative) in Oo was still positive (negative) in 2C. We found that 16% (8/49) of Class A and 83% (35/42) of Class B genes had increased activities in 2C compared with Oo. (D) Among 150 TFBSs (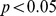), 98 TFBSs (Class A, upregulated in MEP) and 114 TFBSs (Class B, upregulated in Mk) exhibited significant gains and losses of activity (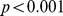). We also found that 83% (81/98) of Class A and 76% (87/114) of Class B genes exhibited no changes in the effects of their TFBS activities. All of the TFBSs in both classes exhibited increases in the strengths of their activities in Mk compared with MEP. *R*, Pearson's correlation coefficient; Oo, MII oocytes; 2C, 2-cell embryo; MEP, megakaryocyte/erythroid precursor; Mk, megakaryocyte.(EPS)

Table S1 **RNA-seq mapping statistics.**
(XLSX)

Table S2 **Differentially expressed cell-surface molecules.**
(XLSX)

Table S3 **Differentially expressed transcription factors.**
(XLSX)

Table S4 **Transcription factors categorized into Class C.**
(XLSX)

Table S5 **Low expressed transcription factors (Class D).**
(XLSX)

Table S6 **Average regression coefficient of 142 TFBSs.**
(XLSX)

Table S7 **Classification of MkE, GM, and Lymphoid-associated genes.**
(XLSX)

Table S8 **TFBSs significantly different in the regression coefficient between LT- and ST-HSCs (Class A).**
(XLSX)

Table S9 **TFBSs significantly different in the regression coefficient between LT- and ST-HSCs (Class B).**
(XLSX)

Table S10 **Enriched GO terms in Class A.**
(XLSX)

Table S11 **Enriched GO terms in Class B.**
(XLSX)

Table S12 **Result of log-linear model in Class A.**
(XLSX)

Table S13 **Result of log-linear model in Class B.**
(XLSX)
